# Microbiology and Outcome of Peritoneal Dialysis-Related Peritonitis in Elderly Patients: A Retrospective Study in China

**DOI:** 10.3389/fmed.2022.799110

**Published:** 2022-04-14

**Authors:** Panai Song, Dong Yang, Jine Li, Ning Zhuo, Xiao Fu, Lei Zhang, Hongqing Zhang, Hong Liu, Lin Sun, Yinghong Liu

**Affiliations:** Department of Nephrology, The Second Xiangya Hospital of Central South University, Hunan Key Laboratory of Kidney Disease and Blood Purification, Changsha, China

**Keywords:** elderly patient, peritoneal dialysis, peritonitis, microbiology, outcome

## Abstract

**Objective:**

The number of elderly patients on peritoneal dialysis (PD) has rapidly increased in the past few decades. We sought to explore the microbiology and outcomes of peritonitis in elderly PD patients compared with younger PD patients.

**Methods:**

We conducted a retrospective study to analyze the clinical characteristics, causative organism distribution, and outcome of all PD patients who developed peritonitis between September 1, 2014 and December 31, 2020, from Second Xiangya Hospital, Central South University, China. Patients who experienced peritonitis were separated into elderly and younger groups. The elderly was defined as ≥ 65 years old at the initiation of PD.

**Results:**

Among 1,200 patients, 64(33.9%) in elderly (*n* = 189) and 215 (21.3%) in younger (*n* = 1,011) developed at least one episode of peritonitis. A total of 394 episodes of peritonitis occurred in 279 patients. Of these, 88 episodes occurred in 64 elderly patients, and 306 episodes occurred in 215 younger patients. Gram-positive bacteria were the main causative organisms in elderly and younger patients (43.2% and 38.0%, respectively). Staphylococcus and Escherichia coli were the most common gram-positive and gram-negative bacteria, respectively. Fungal peritonitis in elderly patients was higher compared with younger patients (χ2 = 6.55, *P* = 0.01). Moreover, Acinetobacter baumannii (χ ^2^=9.25, *P* = 0.002) and polymicrobial peritonitis (χ ^2^ = 6.41, *P* = 0.01) in elderly patients were also significantly higher than that in younger patients. Additionally, elderly PD patients had higher peritonitis-related mortality than younger patients (χ ^2^ = 12.521, *P* = 0.000), though there was no significant difference in catheter removal between the two groups. Kaplan-Meier analysis showed that cumulative survival was significantly lower in elderly patients than younger patients (log rank = 7.867, *p* = 0.005), but similar technical survival in both groups (log rank = 0.036, *p* = 0.849).

**Conclusions:**

This retrospective study demonstrated that elderly PD patients were more likely to develop Acinetobacter baumannii, fungal and polymicrobial peritonitis than younger PD patients. In addition, peritonitis-related mortality was significantly higher in elderly patients, whereas peritonitis-related catheter removal was comparable between elderly and younger PD patients. Understanding microbiology and outcome in elderly patients will help to reduce the incidence of PD-associated peritonitis and improve the quality of life.

## Introduction

With the aging of the world's population, the number of elderly patients with end-stage renal disease increase (1, 2). Peritoneal dialysis (PD) is a vital kidney replacement therapy. It offers a number of potential advantages for elderly patients, including better cardiovascular stability due to slower, continuous ultrafiltration, no need for vascular access, fewer technical requirements, better autonomy and independence, and less disturbing for patient's daily life (3, 4).

Peritonitis is still a common and severe complication of PD (5). Comparisons of risk of peritonitis and outcomes between older and younger patients on PD rely on related studies. However, the results of these studies are variable. It has been hypothesized that elderly patients are at increased risk of peritonitis due to immunocompromise and malnutrition (6). Some studies have reported that older age was independently associated with higher peritonitis (7, 8). In addition, published studies observed no significant difference between elderly and younger patients in cumulative patient survival and technique survival (9–12). Another study reported that elderly patients (>65 years) had a higher peritonitis-related and all-cause mortality, but a similar peritonitis-free survival and a lower risk of death-censored technique failure compared with younger patients (<50 years) (13). The microbiology of peritonitis in elderly PD patients has also rarely been reported previously.

Here, we did a retrospective cohort study aimed to compare the microbiology and outcomes of peritonitis between elderly (≥ 65 years old) and younger (< 65 years old) patients receiving continuance ambulatory peritoneal dialysis (CAPD).

## Materials and Methods

### Study Population

**Figure 1:**
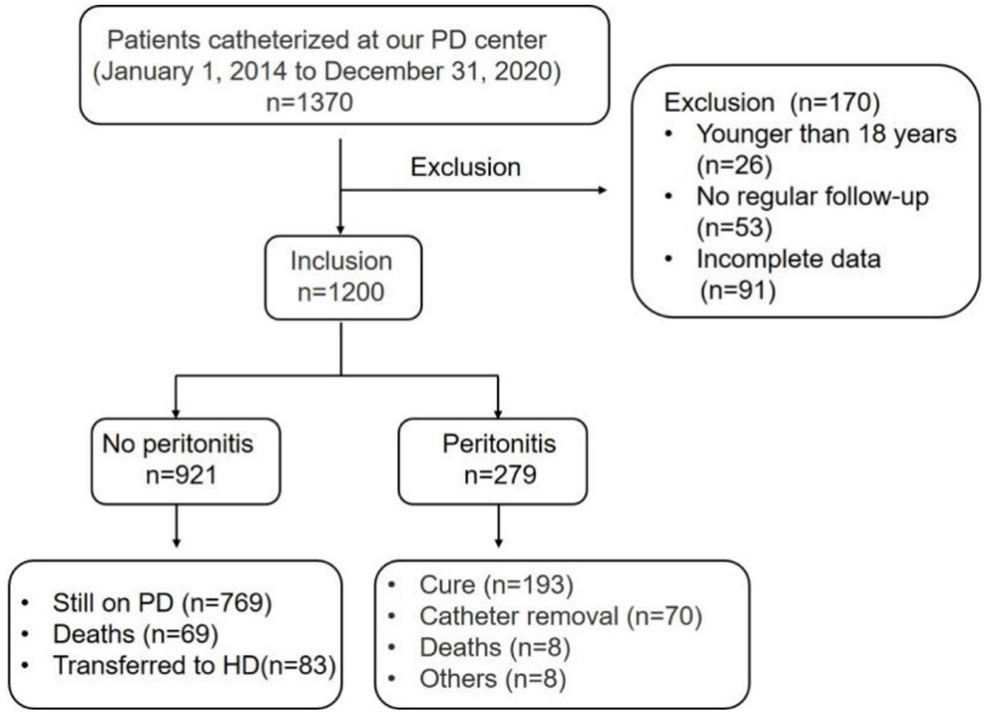
Flow chart for the study participants enrollment and outcomes.

A total of 1,370 patients received PD therapy in our PD center between 1 January 2014 and 31 December 2020. Patients who were younger than 18 years (*n* = 26), with no regular follow-up (*n* = 53), and with incomplete data (*n* = 91) were excluded. A total of 1200 patients were ultimately included in the study (Figure 1). Among 1,200 patients, 921 were non-peritonitis patients and 279 were peritonitis patients. 279 patients developed 394 peritonitis episodes. Inclusion criteria of peritonitis: CAPD patients who met the diagnosis of peritonitis as defined in the ISPD peritonitis recommendations (14). Patients were divided into two groups: elderly patients (≥65 years old) and younger patients (<65 years old). All patients underwent standard laparotomy, using standard double-cuff Tenckhoff straight tubes, and all patients were treated with “Y” type tubes and dialysate produced by Baxter, USA. Approval of study by the research ethics committee of Second Xiangya Hospital, Central South University, and informed consents were provided before their inclusion in the study.

### The Diagnosis of Peritonitis

The diagnosis of PD-associated peritonitis follows the 2016 Guidelines of the International Peritoneal Dialysis Association: (1) clinical features are consistent with peritonitis, with abdominal pain or cloudy dialysis effluent; (2) dialysis effluent white cell count >0.1 × 10^9^ /L(after a dwell time of at least 2 h), with 50% neutrophils; and (3) positive dialysis effluent culture. The diagnosis of PD-associated peritonitis requires any two of the following features (14). The specific methods are as follows: when the patient comes to our outpatients department, dialysis effluent (at least stay in the abdominal cavity for 2 h or more) was taken for regular biochemistry detection and bacteria culture, peritoneal lavage was given immediately once the diagnosis of peritonitis, and empirical antibiotic therapy was initiated as soon as possible after microbiological specimens have been obtained. We usually selected vancomycin combined with third-generation cephalosporin or etimicin covering both gram-positive and gram-negative organisms, and antibiotic therapy was adjusted to narrow-spectrum agents based on culture and sensitivity results. The course of treatment was about 2–3 weeks. Once fungi are identified in PD effluent, catheter removal immediately, and anti-fungal agent was continued for at least 2 weeks after catheter removal.

### Methods of Causative Organism Culture and Drug Sensitivity

Organism identification and antibiotic sensitivities help to guide the choice of antibiotic. 10–20 ml PD effluent was taken from the bottom of the dialysis bag after precipitation with a syringe and directly inoculated into aerobic and anaerobic blood culture bottles. The samples were cultured by BACTEC FX200 automatic blood culture instrument from BD company, USA. Culture medium in the bottle with positive blood culture was inoculated onto blood plate and MacConkey plate for further isolation and culture until a single pure colony was obtained. Finally, identification and drug sensitivity tests were completed by BDPHOENEX100 analyzer.

### Relevant Definitions

Peritonitis-related mortality: It was recorded if the patient's death was deemed directly attributable to peritonitis in the clinical opinion of the treating nephrologist (15). Cure: Peritonitis is wholly relieved after treatment, and there is no need for other anti-infection therapy or removal of the catheter and no recurrence within 30 days. Catheter removal: Because of refractory, relapsing, or fungal peritonitis, PD catheters were removed from the patients. Polymicrobial peritonitis: refers to multiple organisms (multiple gram-positive, multiple gram-negative, or mixed gram-negative/gram-positive organisms) are grown from PD effluent. Dropout: Patients with peritonitis were transferred to hemodialysis after catheter removal, withdrawing treatment, or patients who have not been completely cured but have been lost to follow-up due to various reasons.

### Statistical Analysis

SPSS25.0 software was used for statistical analysis, mean ± standard deviation or median and range, as appropriate. Student's *t*-test was used to compare normal distribution data sets, and Wilcoxon rank-sum test was used to compare non-normal distribution data sets. Statistical data were presented by composition ratio or rate, Chi-square test or Fisher exact test were used for comparison, and Kaplan-Meier method was used to calculate cumulative survival rate and draw survival curve, and Log-rank method was used to test differences between groups. Values of *P* < 0.05 were considered statistically significant.

## Results

### Patients Characteristics

Among 1,200 patients, 64(33.9%) in elderly (*n* =189) and 215 (21.3%) in younger (*n* = 1,011) developed at least one episode of peritonitis during this period. A total of 394 episodes of peritonitis occurred in 279 patients. Of these, 88 episodes occurred in 64 elderly patients and 306 episodes occurred in 215 younger patients. The median ages of the elderly and younger patients at dialysis initiation were 72 and 48 years old, respectively. Detailed information about patient characteristics is provided in Table 1. The baseline demographic data and clinical parameter of the two study groups were comparable except that the elderly PD patients had a significantly higher incidence of diabetes mellitus, hypertension and obstructive nephropathy and had a lower baseline serum albumin, serum creatinine, uric acid, and serum phosphorus than the younger PD patients (Table 1).

**Table 1:** Baseline characteristic of the study population.

	**Elderly group (*n* = 64, 88 episodes)**	**Younger group (*n* = 215, 306 episodes)**	**χ2/F**	***P***
Gender (male)	57	170	2.493	0.114
Age (year)	71.6 ± 4.86	47 ± 10.362	45.69	0.000^*^
Dialysis vintage (month)	36.98 ± 3.51	30.29 ± 1.60	1.418	0.28
BMI(kg/m^2^)	22.51 ± 3.13	21.89 ± 3.13	0.178	0.324
**Primary disease**
Chronic glomerulonephritis	33 (37.5%)	194 (63.4%)	25.738	0.000^*^
Diabetic nephropathy	15 (17%)	24 (7.8%)	6.489	0.011^*^
Hypertensive nephropathy	25 (28.4%)	55 (18%)	4.599	0.032^*^
Obstructive nephropathy	12 (13.6%)	3(1%)	29.892	0.000^*^
Other	3(3.2%)	25(8.4%)		
**Laboratory findings, mean** **±SD**
Hemoglobin, g/L	98.52 ± 22.81	100.38 ± 20.13	0.727	0.652
Albumin, g/L	33.11 ± 3.97	34.99 ± 5.36	4.599	0.032^*^
BUN, mmol/L	18.36 ± 6.65	20.79 ± 7.11	0.506	0.08
Creatinine, mmol/L	680.2 ± 308.2	889.5 ± 312.0	0.823	0.001^*^
Uric acid, mmol/L	387.5 ± 53.6	445.2 ± 99.4	7.424	0.000^*^
TCHO, mmol/L	4.91 ± 1.21	4.87 ± 1.20	0.013	0.875
LDL, mmol/L	3.08 ± 1.02	3.02 ± 0.89	0.601	0.744
Calcium, mmol/L	2.05 ± 0.23	2.07 ± 0.23	0.023	0.617
Phosphorus, mmol/L	1.47 ± 0.49	1.71 ± 0.48	0.208	0.012^*^
GFR (mL/min/1.73 m^2^)	3.57 ± 2.68	3.41 ± 2.57	0.438	0.756
iPTH, mmol/L	23.75 ± 16.28	32.26 ± 24.31	3.705	0.025^*^
Total Kt/V	1.81 ± 0.64	2.10 ± 0.93	0.709	0.111
nPCR (g/kg/day)	1.28 ± 0.31	1.58 ± 0.50	1.671	0.119

*Age, defined as age at the initiation of PD; BMI, body mass index; TCHO, Total cholesterol; LDL, low density lipoprotein; PTH, parathyroid hormone; GFR, glomerular filtration rate; nPCR, normalized catabolic rate*.

* *P < 0.05*.

### Microbiology Distribution

The microbiology distribution of peritonitis was listed in Table 2. There were 38 peritonitis episodes were gram-positive bacteria (43.2%), 26 episodes were gram-negative bacteria (29.5%), and 12 episodes were fungi (13.6%) in the elderly group. And 116 peritonitis episodes were gram-positive bacteria (38.0%); 69 episodes were gram-negative bacteria (22.6%); 17 episodes were fungi (5.6%) in the younger group. Gram-positive bacteria were the primary causative organism in both elderly and younger patients (43.2 and 38.0%, respectively). Fungal peritonitis was significantly higher in the elderly group than younger patients (χ^2^ = 6.55, *P* = 0.01), but there is no significant difference in the proportions of gram-positive and gram-negative bacteria between the two groups (*P* = 0.38 and 0.18, respectively).

**Table 2:** Causative organisms distribution in elderly and younger patients with PD related peritonitis.

**Strains**	**Elderly group** ***n*** **=** **88**		**Younger group** ***n*** **=** **306**		**χ2**	***P***
	**Episodes**	**Ratio%**	**Episodes**	**Ratio%**		
G+ bacteria	38	43.20%	116	38.00%	0.76	0.383
Staphylococcus	27	30.70%	69	22.50%	2.453	0.117
Coagulase-negative staphylococcus	12	13.60%	34	11.10%	0.423	0.516
*Staphylococcus epidermidis*	3	3.40%	17	5.60%	0.654	0.419
*Staphylococcus hemolyticus*	2	2.30%	3	1.10%	0.911	0.340
*Staphylococcus aureus*	10	11%	30	9.80%	0.182	0.669
Streptococcus	8	9.10%	32	10.50%	0.14	0.708
*Hemolytic streptococcus*	3	3.40%	19	6.20%	1.016	0.313
*Streptococcus salivarius*	1	1.10%	8	2.60%	0.669	0.413
Enterococcus	1	1.10%	7	2.30%	0.455	0.500
G- bacteria	26	29.50%	69	22.60%	1.786	0.181
*Escherichia coli*	8	9.10%	31	10.10%	0.083	0.773
*Acinetobacter baumannii*	7	8.00%	5	1.60%	9.246	0.002^*^
*Klebsiella pneumoniae*	4	4.50%	9	2.90%	0.551	0.458
*Pseudomonas aeruginosa*	2	2.30%	6	2.00%	0.033	0.855
Neisseria	0	0.00%	4	1.30%	1.162	0.281
Fungus	12	13.60%	17	5.60%	6.545	0.011^*^
*Candida albicans*	6	6.80%	6	2.00%	5.461	0.019^*^
Saccharomycetes	1	1.14%	2	0.65%	–	–
Polymicrobial infection	7	8.00%	7	2.30%	6.405	0.011^*^
Culture negative	19	22.60%	107	35.00%	4.688	0.030^*^

* *P < 0.05*.

With analysis of gram-positive bacteria, *Staphylococcus* was the most common bacteria in peritonitis cases, the proportions of *Staphylococcus, Streptococcus*, and *Enterococcus* in both elderly and younger patients were similar. The most common gram-negative bacteria were *Escherichia coli*, followed by *Acinetobacter baumannii, Klebsiella pneumoniae, Pseudomonas aeruginosa*. The proportion of *Acinetobacter baumannii* in elderly patients was significantly higher than in younger patients (χ^2^ = 9.25, *P* = 0.002).

### Classification of Causative Organism and Outcome

According to the results of causative organism culture, peritonitis episodes were divided into single gram-positive bacteria, single gram-negative bacteria, single fungi, polymicrobial infection, and culture-negative peritonitis. The incidence of single gram-positive bacteria and gram-negative bacteria peritonitis were similar in both groups. However, fungal peritonitis in the elderly was higher than in younger patients, although the difference was not statistically significant (*P* = 0.14). Polymicrobial peritonitis (χ ^2^ = 6.41, *P* = 0.01) in elderly patients was also significantly higher but culture-negative peritonitis was lower compared with younger patients (χ ^2^ = 4.69, *P* = 0.03), as shown in Table 3.

**Table 3:** Classification of causative organisms culture in elderly and younger patients with peritonitis.

**Peritonitis**	**Elderly group** ***n*** **=** **88**		**Younger group** ***n*** **=** **306**		**χ2**	***P***
	**Episodes**	**Ratio %**	**Episodes**	**Ratio%**		
Single G+ bacteria	33	37.50%	113	37.00%	0.006	0.939
Single G- bacteria	21	24%	64	21.00%	0.334	0.563
Single fungus	8	9.10%	15	4.90%	2.182	0.140
Polymicrobial infection	7	8.00%	7	2.30%	6.405	0.011^*^
Culture negative	19	22.60%	107	35.00%	4.688	0.030^*^

* *P < 0.05*.

A total of 309 episodes (78.4%) out of 394 episodes achieved medical cure in this study. Failure to achieve medical treatment in 85 episodes was due to 1 or more of the following reasons: catheter removal (70 episodes), peritonitis-related death (8 episodes), and lost to follow-up (8 episodes). The cure rates of elderly and younger patients were 72.70% and 80.10%, the catheter removal rates were 19.30% and 17.30%, and the mortality rates were 6.80 and 0.70%, respectively. The cure rate of peritonitis in elderly patients was lower than that in younger patients, but the difference was not statistically significant (χ^2^ = 2.18, *P* = 0.14). In contrast, the peritonitis-related mortality in elderly patients was significantly higher compared with younger patients (χ^2^ = 12.521, *P* = 0.000402). There was no significant difference in catheter removal between the two groups, as shown in Table 4. In univariate Cox regression analysis, when age, sex, hemoglobin, phosphorus, calcium, albumin, 24-h urine volume, diabetes and cardiovascular disease (CVD) were chosen for adjustment in multivariate Cox proportional-hazards model, the risk of death among elderly PD patients was 2.54 times higher than that in the younger PD patients with a statistically significant difference (HR = 2.538, 95%CI [1.022, 6.305], *P* = 0.045, Table 5). However, after adjusting for age, sex, hemoglobin, phosphorus, calcium, albumin, 24-h urine volume, diabetes and CVD, there was no significant difference in cure between the elderly and younger groups (HR = 1.108, 95%CI [0.437, 2.807], *P* = 0.829, Table 5).

**Table 4:** Outcomes of peritoneal dialysis-associated peritonitis in elderly and younger patients.

**Outcome**	**Elderly group** ***n*** **=** **88**		**Younger group** ***n*** **=** **306**		**χ2**	**P**
	**Episodes**	**Ratio %**	**Episodes**	**Ratio%**		
Cure	64	72.70%	245	80.10%	2.175	0.140
Catheter removal	17	19.30%	53	17.30%	0.187	0.666
Death^a^	6	6.80%	2	0.70%	13.057	0.000^*^
Others^b^	1	1.10%	7	2.30%	0.455	0.50

a *Peritonitis related mortality*;

b *Patients who have not been completely cured but have been lost to follow-up due to various reasons*.

* *P < 0.05*.

**Table 5:** Cox proportional-hazards model for cure and mortality of PD related peritonitis.

**Variable**	**Univariate Cox regression analysis**		**Multivariate Cox regression analysis**	
	**HR [95%CI]**	***P* value**	**HR [95%CI]**	***P* value**
**Cure**				
Age (>65 or <65years)	0.695 [0.318, 1.519]	0.362	1.108 [0.437, 2.807]	0.829
Sex (male)	1.253 [0.792, 1.982]	0.336	1.540 [0.928, 2.554]	0.095
Hemoglobin (g/L)	1.019 [0.647, 1.605]	0.936	0.808 [0.484, 1.350]	0.481
Calcium (mmol/L)	0.603 [0.335, 1.086]	0.092	0.360 [0.173, 0.747]	0.006^*^
Phosphorus (mmol/L)	0.874 [0.531, 1.437]	0.595	1.135 [0.632, 2.036]	0.671
Serum albumin (g/L)	1.389 [0.853, 2.261]	0.187	1.722 [0.963, 3.076]	0.067
Diabetes	0.691 [0.342, 1.394]	0.302	0.392 [0.150, 1.023]	0.056
History with CVD	1.140 [0.583, 2.232]	0.701	1.756 [0.705, 4.373]	0.227
24 h urine volume (ml)	0.725 [0.442, 1.189]	0.203	0.808 [0.484, 1.350]	0.416
**Mortality**				
Age (>65 or <65years)	2.696 [1.226, 5.929]	0.014	2.538 [1.022, 6.305]	0.045
Sex (male)	0.810 [0.384, 1.709]	0.580	0.867 [0.386, 1.950]	0.731
Hemoglobin (g/L)	2.907 [1.287, 6.565]	0.010	2.679 [1.090, 6.586]	0.032^*^
Calcium (mmol/L)	1.379 [0.416, 4.571]	0.600	0.570 [0.148, 2.195]	0.414
Phosphorus (mmol/L)	0.680 [0.319, 1.451]	0.318	1.090 [0.456, 2.607]	0.846
Serum albumin (g/L)	3.452 [1.506,7.913]	0.003	2.001 [0.786,5.097]	0.146
Diabetes	2.399 [1.115, 5.162]	0.025	0.675 [0.246, 1.853]	0.445
History with CVD	3.762 [1.775, 7.973]	0.001	2.720 [1.030, 7.189]	0.044^*^
24 h urine volume (ml)	1.282 [0.545, 3.013]	0.569	0.970 [0.401, 2.346]	0.946

* *P < 0.05*.

### Cumulative Survival and Technical Survival

**Figure 2:**
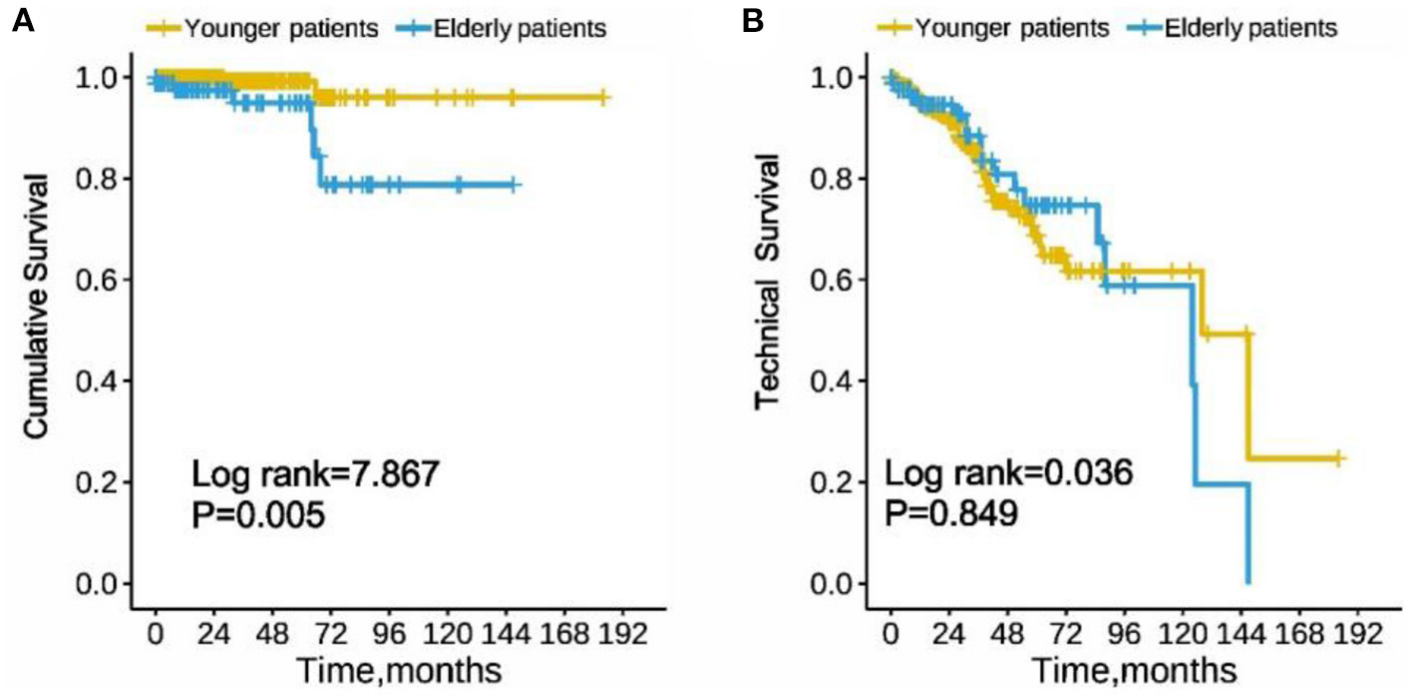
Cumulative survival **(A)** and technique survival **(B)** of elderly and younger patients with peritoneal dialysis-associated peritonitis. (Log-rank test χ2 = 7.867, *p* = 0.005 and Log-rank test χ2 = 0.036, *p* = 0.849, respectively).

Taking peritonitis-related mortality of the CAPD patients as the endpoint, cumulative survival was analyzed. The 1-year, 3-year, and 5-year survival rates of the elderly group were 92, 89, and 82%, respectively, while younger patients were 99, 98, and 94%, respectively. In Kaplan-Meier analysis, the elderly PD group was associated with a significantly lower cumulative survival as compared with the younger group (log rank = 7.867, *p* = 0.005, Figure 2A). However, the technical survival were similar among the two PD groups (log rank = 0.036, *p* = 0.849, Figure 2B).

## Discussion

The current study investigated the microbiology and clinical outcomes of elderly CAPD patients compared with younger CAPD patients. The results showed that *Acinetobacter baumannii* peritonitis in elderly patients was significantly higher than in younger PD patients. And elderly patients were more prone to fungal infection or polymicrobial infection. In addition, the cumulative survival of elderly CAPD patients was inferior to those of younger patients. However, technical survival was similar between elderly and younger CAPD groups.

Peritonitis is a significant complication of PD, and often resulting in the termination of peritoneal dialysis (16). In this study, the primary causative organisms of peritonitis in elderly and younger patients were gram-positive bacteria. *Staphylococcus* predominated for PD-related peritonitis in both groups. The most common gram-negative bacteria was *Escherichia coli*. This bacterial distribution and high incidence of *Staphylococcus* were similar to previous reports (17–20). And such results of the predominance of gram-positive bacteria were similar to the studies in America, Canada, Scotland, and Hong Kong (21–24). The type of causative organisms usually indicates a possible cause of infection. *Coagulase-negative staphylococcal* peritonitis, especially those caused by *Staphylococcus epidermidis*, is mainly caused by contact contamination. *Staphylococcus aureus* infection is also related to contact contamination. Gram-negative bacteria are primarily associated with intestinal infection and constipation (25). This suggests that PD patients, especially elderly patients, should further strengthen the aseptic operation concept and avoid contact infection. In addition, we still need to take positive measures, including enhancing dietary guidance for elderly PD patients, paying attention to regulating intestinal flora, avoiding hypokalemia, improving constipation, and timely treating intestinal infections to reduce the risk of gram-negative bacteria infections in PD patients.

Interestingly, *Acinetobacter baumannii* peritonitis in the elderly group was significantly higher than in the younger group. Several researchers proposed that Acinetobacter peritonitis often occurred with an immunocompromised status (26, 27). Since elderly patients usually have weakened immune systems, they are more susceptible to Acinetobacter infection. *Acinetobacter baumannii* infections are reportedly associated with 10 to 20% higher attributable mortality and longer length of hospital stay (28, 29), owing to their high antibiotic resistance rates (30). The low immunity status also explains the higher mortality rate among elderly patients in the study. Therefore, clinicians should pay more attention to improving the immunity of elderly patients.

Compared with bacterial peritonitis, fungal peritonitis is rare in PD-related peritonitis (31, 32); they are yet bringing terrible damage by higher catheter removal, more extended hospital stay, permanent transfer to hemodialysis, and mortality (14, 33). Candida species are the most typical organism for most fungal peritonitis cases, accountable for about 70–90% of fungal peritonitis in adults (31, 33). Our findings were similar to those of these studies, and fungal infections were higher in elderly patients. Previous studies have shown that malnutrition, diabetes mellitus, prior use of antibiotics, immunocompromised state, and prolonged-time on PD are the main risk factors for fungal peritonitis (32, 34). This study found that elderly PD patients had a higher proportion of *acinetobacter baumannii* peritonitis due to their immunocompromised status, so the antibiotics treatment was intense and prolonged, which led to a high chance of superinfection. And we also found that the prevalence of anemia, hypoproteinemia and diabetes in the elderly patients were higher. Therefore, nephrologists should strengthen the nutritional management of elderly patients, improve their anemia, and actively treat their diabetes.

The incidences of culture-negative peritonitis were 22.6% (19/88 episodes) in elderly patients, 35.0% (107/306 episodes) in younger patients. The relatively high culture-negative proportion in both groups may be primarily related to the early use of antibiotics before admission to our center. In addition, a small part of the reasons may be limited effluent culture technique, although the microbial culture technical in our hospital has been dramatically improved in recent years. Therefore, we should follow the recommendations of the ISPD guideline, such as centrifugation of effluent, incubation in aerobic, microaerophilic, and anaerobic environments, using antibiotic neutralization bottles to improve the yields of effluent organism culture (14, 35).

It is also important to note that the peritonitis-related mortality in elderly peritonitis patients was higher; this may have to do with elderly patients having more comorbidity, more unsatisfactory performance, and was more frequently malnourished. In further multivariate analysis we did find that anemia and cardiovascular comorbidity were independent risk factors for peritonitis-related mortality in elderly PD patients. However, catheter removal has no statistical significance in both groups. Previous studies (36–38) reported that patient survival rate was poorer in the elderly PD patients than in younger PD patients, but similar technical survival. Our study's finding follows that of previous studies, given that there was no significant difference in technical survival between the elderly and younger PD patients. In contrast, cumulative survival was significantly lower in the elderly PD patients than younger patients.

Our study is rare in comparing microbiology distribution and peritonitis outcomes between elderly and younger PD patients. These bright spots should be balanced with the potential limitations, including a single-center, retrospective study with a relatively small number of elderly PD patients, no analysis and discussion of treatment and antimicrobial resistance. Moreover, potential coding bias and other confounding factors cannot be entirely excluded. Therefore, a multi-center, more extensive, long-term, prospective study is needed in the future to confirm our findings.

In conclusion, this retrospective study demonstrated that elderly PD patients were more likely to develop baumannii, fungal, and polymicrobial peritonitis than younger PD patients. In addition, peritonitis-related mortality was significantly higher in elderly patients, whereas peritonitis-related catheter removal was comparable between elderly and younger PD patients. Understanding microbiology distribution and outcome, identifying possible barriers in elderly patients will help to reduce the incidence of PD-associated peritonitis and improve the quality of life.

## Data Availability Statement

The original contributions presented in the study are included in the article/supplementary materials, further inquiries can be directed to the corresponding author/s.

## Ethics Statement

This retrospective study was approved by the Ethics Committee of Second Xiangya Hospital and performed in accordance with the Declaration of Helsinki and informed consents were provided before the participants' inclusion in the study.

## Author Contributions

PS collected clinical data, drafted, and revised manuscript. JL, DY, and HZ collected clinical data and searched the relative literatures. NZ searched the relative literatures, made analysis, and revised the English of manuscript. XF, LZ, HL, and LS provided with clinical assistance and contributed to the acquisition of these data. YL revised the manuscript and takes responsibility for the work, all authors have read and approve of the final version. All authors contributed to the article and approved the submitted version.

## Funding

This work was supported by National Natural Science Foundation of China (81800649), Changsha Municipal Natural Science Foundation (kq2202401, kq2014235), and Natural Science Foundation of Hunan Province (2021JJ30942).

## Conflict of Interest

The authors declare that the research was conducted in the absence of any commercial or financial relationships that could be construed as a potential conflict of interest.

## Publisher's Note

All claims expressed in this article are solely those of the authors and do not necessarily represent those of their affiliated organizations, or those of the publisher, the editors and the reviewers. Any product that may be evaluated in this article, or claim that may be made by its manufacturer, is not guaranteed or endorsed by the publisher.
